# Assessing the reliability and cross-sectional and longitudinal validity of fifteen bioelectrical impedance analysis devices

**DOI:** 10.1017/S0007114522003749

**Published:** 2023-09-14

**Authors:** Madelin R. Siedler, Christian Rodriguez, Matthew T. Stratton, Patrick S. Harty, Dale S. Keith, Jacob J. Green, Jake R. Boykin, Sarah J. White, Abegale D. Williams, Brielle DeHaven, Grant M. Tinsley

**Affiliations:** Department of Kinesiology and Sport Management, Texas Tech University, Lubbock, TX, USA

**Keywords:** Bioimpedance, Body composition, Body fat, Bioelectrical impedance analysis

## Abstract

The purpose of this investigation was to expand upon the limited existing research examining the test–retest reliability, cross-sectional validity and longitudinal validity of a sample of bioelectrical impedance analysis (BIA) devices as compared with a laboratory four-compartment (4C) model. Seventy-three healthy participants aged 19–50 years were assessed by each of fifteen BIA devices, with resulting body fat percentage estimates compared with a 4C model utilising air displacement plethysmography, dual-energy X-ray absorptiometry and bioimpedance spectroscopy. A subset of thirty-seven participants returned for a second visit 12–16 weeks later and were included in an analysis of longitudinal validity. The sample of devices included fourteen consumer-grade and one research-grade model in a variety of configurations: hand-to-hand, foot-to-foot and bilateral hand-to-foot (octapolar). BIA devices demonstrated high reliability, with precision error ranging from 0·0 to 0·49 %. Cross-sectional validity varied, with constant error relative to the 4C model ranging from −3·5 (sd 4·1) % to 11·7 (sd 4·7) %, standard error of the estimate values of 3·1–7·5 % and Lin’s concordance correlation coefficients (CCC) of 0·48–0·94. For longitudinal validity, constant error ranged from −0·4 (sd 2·1) % to 1·3 (sd 2·7) %, with standard error of the estimate values of 1·7–2·6 % and Lin’s CCC of 0·37–0·78. While performance varied widely across the sample investigated, select models of BIA devices (particularly octapolar and select foot-to-foot devices) may hold potential utility for the tracking of body composition over time, particularly in contexts in which the purchase or use of a research-grade device is infeasible.

The body composition analysers market is expected to demonstrate a compound annual growth rate of 7·2 % by 2027, with bioelectrical impedance analysis (BIA) devices contributing a large share of this growth^(1)^. In addition to their range of applications across clinical, health and wellness, research and home use settings, BIA devices boast relative convenience and affordability compared with other methods of body composition assessment such as dual-energy X-ray absorptiometry (DXA) or air displacement plethysmography (ADP). In recent years, numerous studies have examined the validity and reliability of commonly used BIA devices for the assessment of body composition in a variety of populations. However, these investigations commonly utilise standalone body composition assessment techniques such as ADP^(2,3)^, DXA^(4–9)^ or a research-grade BIA device^(10)^ as the chosen reference method as opposed to a four-compartment (4C) model^(11–14)^, which takes into account the estimated bone mineral content, total body water and body volume from the most valid assessment techniques available. In addition, although some of the devices used in previous research are consumer-grade (i.e. available to consumers for home use at a relatively low price-point, such as within approximately $20–350 USD), many studies also often include or are limited to relatively high-cost or research-grade devices which are not designed for home use and are thus of less relevance to most consumers.

Furthermore, the great majority of research examining the characteristics of consumer-grade BIA devices has focused on the cross-sectional validity of these devices, but fewer investigations have assessed their test–retest reliability or their longitudinal validity (i.e. their ability to accurately assess changes in body composition over time). This represents an important gap in the literature, as changes in a given estimate of body fat or fat-free mass over time – particularly in response to an exercise or nutrition intervention – are likely of more interest to the typical consumer of a home BIA device than the validity of the given estimate at a singular time point. Finally, while some studies have examined the validity and/or reliability of multiple research and consumer-grade BIA devices, these investigations are typically limited to no more than five models, limiting the breadth and applicability of findings.

Thus, the purpose of this study was to examine both the cross-sectional and longitudinal validity and the test–retest reliability of a wide variety of consumer-grade BIA devices designed for home use and available within a range of price-points (from approximately $24 to $349 USD at the time of purchase) using a laboratory 4C model as the criterion method. In addition to the presentation of findings, best-practice suggestions are provided for the use of commercially available BIA devices to track changes in body fat percentage (BFP) over time.

## Materials and methods

### Participants

Generally, healthy adults between 18 and 50 years of age who had maintained a relatively stable body weight (i.e. no more than a gain or loss of approximately 2·3 kg^(15)^ based on self-report) for the past month at the time of enrolment were recruited to participate. Individuals who reported any body composition abnormality, amputation, physical deformity large enough to invalidate results from any of the devices, pacemaker or other electrical implant, beard longer than approximately 1·3 cm or large amount of body hair (due to the effect of excess facial or body hair on the validity of body volume measurements via ADP^(16)^), height (up to 192 cm) exceeding the scan zone of the DXA device or weight (up to 150 kg) exceeding the capacity of any of the devices were excluded from participation. However, individuals with a history of liposuction or of the implantation of a small amount of metal (e.g. rods, screws) or non-metal devices (e.g. breast augmentation) were included in the sample and their history of these procedures documented accordingly.

### Ethical approval

This study was conducted according to the guidelines laid down in the Declaration of Helsinki, and all procedures involving human subjects were approved by the Texas Tech University institutional review board (IRB2021–107). Written informed consent was obtained from all subjects. Plans for data collection were also registered prospectively at clinicaltrials.gov (Clinical Trials.gov identifier: NCT05026697).

### Pre-assessment standardisation and intake

The study consisted of a maximum of two visits: one initial visit and an optional follow-up visit scheduled 12–16 weeks later. All participants were instructed to abstain from exercise and vigorous physical activity for 24 h and to abstain from all food, fluid, caffeine, alcohol, nicotine or other substances for 8 h prior to each scheduled visit. To support adequate hydration during the visit, participants were also instructed to ingest 1 litre of water between their last meal and the beginning of the 8-h abstention from fluid. To further standardise measurements, participants wore skin-tight clothing (e.g. compression shorts, sports bra for females) for the duration of each visit.

All visits were scheduled to commence between the hours of 6.00 and 14.00. Upon arrival to the laboratory during the initial visit, participants were screened to confirm eligibility, the investigation was verbally explained to participants and written informed consent was obtained. At each visit, participants’ adherence to the pre-testing guidelines was confirmed. Participants were then instructed to void their bladder. At this time, a urine sample was collected for the assessment of urine specific gravity using a digital refractometer (PA201X-093, Misco). Participants were then instructed to remove their shoes, socks, any additional clothing and all metal jewellery and other accessories before proceeding with all body composition assessments.

### Anthropometric assessment and bioelectrical impedance analysis

All devices requiring calibration were calibrated daily before visits in accordance with manufacturer instructions. First, body mass was assessed with the calibrated scale associated with the ADP device (Model BWB-627-A, modified Tanita Corp.) and height measured to the nearest 0·1 cm using a stadiometer (HM200P, Charder Medical). Then, after participants were provided with a swim cap to collect and cover all hair, anthropometric values were collected with a three-dimensional optical scanner (SS20, Size Stream, scanner version 6.2, software version 5.2.7 for Size Stream Studio). After participants had maintained an upright, standing position for a minimum of 10 min (including the initial anthropometric assessments), participants’ BFP estimates were then collected from each of fifteen BIA devices in a predetermined, randomised order. The assessment order for each participant was generated using the *sample* randomisation function within R software. Participants were further instructed to continue standing throughout the duration of the BIA assessments.

The sample of BIA devices included fourteen consumer-grade models in addition to one research-grade model. The sample comprised one hand-to-hand device (device A); ten foot-to-foot devices (devices B–H and J–L) and four hand-to-foot, bilateral, octapolar models, including three consumer-grade devices (devices I, M and N) and one research-grade model (O). In order to obtain a sample representing a wide array of price-points and configurations, devices that were highly popular among online consumers at the time of purchase (i.e. best-selling items on Amazon.com) were chosen in addition to select models produced by body composition, health and home device manufacturers known to the authors. Details regarding the design and use of each model can be found in online Supplement 1. For each device, participant height was entered to the highest precision allowed by the device (up to the nearest 0·1 cm) and rounded to the nearest 0·5 or 1·0 cm as needed. When participant birthday was required in lieu of age, this was entered as the first day of January of the participant’s birth year to avoid use of protected health information. If activity level was required, this was entered as the lowest possible setting (e.g. ‘1’/sedentary or ‘normal’ mode) to promote standardisation across individuals and devices.

For each of the four octapolar models, participants were instructed on proper hand and arm placement and then provided the handle(s) if needed once standing on the device. During the initial visit, estimates from each device were collected twice in succession to assess test–retest reliability. For these assessments, participants stepped off or away from each device (i.e. fully reset their foot and/or hand positioning) before immediately retesting. The first of these two readings was used to assess cross-sectional validity compared with the laboratory 4C model. During an optional follow-up visit, singular estimates of BFP were recorded from each device in the same randomised order as the initial visit to assess the longitudinal validity of each device. Participants were not specifically instructed to change any aspect of their habitual lifestyle after their initial visit but were told that they could lose, gain or maintain weight between visits.

### Four-compartment model

A laboratory 4C model was used to assess both the cross-sectional and longitudinal validity of the BFP estimates obtained from each device. This model included body mass obtained via calibrated scale and body volume assessed via ADP (BodPod, Cosmed USA), bone mineral content obtained from DXA (Lunar Prodigy, General Electric, with enCORE software version 16.2) and total body water assessed via bioelectrical impedance spectroscopy (SFB7, Impedimed) using methods in accordance with manufacturer instructions and as previously described in detail elsewhere^(17)^. However, rather than taking the average of duplicate total body water values, simply the first value was used in line with the methods applied across all BIA devices. When participants were too broad to fit within the designated scan zone of the DXA device, a reflection scan was used to estimate excluded limbs. Previous research has shown this method to introduce minimal error^(18,19)^. In addition, research staff manually adjusted region of interest lines within the enCORE software to delineate each body segment (i.e. head, trunk and limbs). Resulting bone mineral content estimates were multiplied by 1·0436 to generate a value for bone mineral (Mo). To obtain a 4C BFP value, the Wang 2002^(20)^ equation was used, as shown:

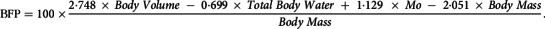




### Statistical analysis

Statistical analyses were conducted in R (version 4.1.2). Values of interest for the assessment of test–retest reliability included precision error (PE; calculated as 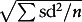 , where sd is the within-subject standard deviation), least significant change (calculated as 2·77 × PE to reflect a 95 % confidence level), mean difference (calculated as the mean of within-participant differences between first and second BFP estimates during the initial visit) and the maximum absolute difference (defined as the absolute value of the maximum difference between the first and second BFP estimates across participants during the initial visit). For the assessment of cross-sectional validity, constant error was calculated as the mean of the individual differences between the BFP estimate of each BIA device and that of the criterion 4C model, and total error was calculated as the root mean square error between the estimate of each BIA device and that of the 4C model. In addition to null hypothesis significance testing with 95 % confidence limits, 90 % confidence limits for two one-sided *t* tests were used to assess whether each BIA device demonstrated equivalence with the criterion 4C model such that the entire confidence interval needed to be contained within the equivalence region in order for the values observed by the BIA device to be considered equivalent to the estimates of the 4C model^(21)^. While a ± 2 % equivalence region was used for assessment of cross-sectional validity in line with previous work^(17)^, a ± 1 % equivalence region was used for longitudinal validity due to the small mean change (+0·2 %) observed in the 4C estimate. Both of these methods were conducted using the *TOSTER* package in R^(22)^. Additionally, Bland–Altman analysis was performed^(23)^, including generation of the 95 % limits of agreement and linear regression to allow for examination of proportional bias.

Deming regressions^(24)^, which account for the error produced within both the criterion (4C) and the alternate (BIA) device estimates, were used in addition to ordinary least squares (OLS) models, which accounts only for error within one method. Both of these methods compare the intercept and slope of regression lines to the line that would be produced if there were a perfect relationship between the estimates (i.e. an intercept of 0 and a slope of 1). For both analyses, the 4C model was chosen for the criterion (*y*-axis) variable with the comparison BIA model designated as the predictor (*x*-axis) variable. Standard error of the estimate was defined as the residual standard error value from OLS regression. Other values of interest included Lin’s concordance correlation coefficient (CCC), Pearson’s correlation coefficient (*r*) and the coefficient of determination (*R* ^2^). Values of interest for longitudinal validity generally mirrored those for cross-sectional validity and compared the estimates of change in BFP over time from each BIA device to the changes observed in the 4C model. Values are presented as mean values and standard deviations, and statistical significance was accepted at *P* < 0·05.

Due to the exploratory nature of this analysis, sample size was based on resource availability; however, the resulting sample size was comparable to or larger than several other investigations within the literature^(2,3,5–7,11–13)^.

### Performance scoring

To enable interpretation of the performance of each device within a range of domains, a ranking system was utilised. Scores for test–retest reliability were based on the PE of the device. Cross-sectional and longitudinal group validity scores were based on the total error of the device, whereas the 95 % limits of agreement on the Bland–Altman plot were used to rank devices for individual-level cross-sectional and longitudinal validity. Devices were then scored from 1 (best performer) to 15 (worst performer) within each domain, with smaller scores indicating better performance, and devices tied for the same place if necessary. Scores from each of the five domains were then summed to result in a global performance score from best (lowest score) to worst (highest score) and given a final rank of 1–15.

## Results

### Participants

A total of seventy-three participants (thirty-eight females and thirty-five males) were included in the analysis of reliability and cross-sectional validity using data from their initial visit. The sample included three participants (4 %) who identified as African American/Black, ten (14 %) who identified as Asian, forty-one (56 %) who identified as White/Caucasian, eighteen (25 %) who identified as Hispanic and one (1 %) who identified as Native Hawaiian/other Pacific Islander. Of the thirty-eight females who were included in the data for the initial visit, twenty-nine (76 %) reported having a regular menstrual cycle, defined as menstrual periods that occur at predictable intervals and no missed periods in the past 6 months. Twenty-three (61 %) reported being on some form of hormonal contraception. Of these, twelve (52 %) reported using the combined oral contraceptive pill, two (9 %) reported using a progestin-only oral contraceptive pill, seven (30 %) reported using a hormonal intra-uterine device and one (4 %) each reported using a hormonal implant or vaginal ring.

Of the seventy-three total participants, thirty-seven (sixteen females and twenty-one males) returned for an optional follow-up visit 12–16 weeks (mean: 89·2 (sd 5·8) d or 12·7 (sd 0·8) weeks; range: 84–104 d or 12·0–14·9 weeks) after their initial assessment and were included in the analysis of longitudinal validity using data from both visits. Of this sample, one (3 %) identified as African American/Black, seven (19 %) identified as Asian, twenty (54 %) identified as White/Caucasian, eight (22 %) identified as Hispanic and one (3 %) identified as Native Hawaiian/other Pacific Islander. Of the sixteen females who returned for a follow-up visit, sixteen (100 %) reported a regular menstrual cycle at the time of their second visit. Ten (63 %) reported current use of hormonal contraception. Of these, five (50 %) reported use of a combined oral contraceptive pill and two (20 %) reported use of a progestin-only oral contraceptive pill. Meanwhile, three (30 %) reported use of a hormonal intra-uterine device, and none of the participants reported the use of a hormonal implant or vaginal ring. Baseline characteristics for the entire sample of participants, as well as for the subset who returned for a follow-up visit, are presented in Table 1.

**Table 1.** Participant baseline characteristics

	Age (years)		Height (cm)		Weight (kg)		4C BFP (%)		BMI (kg/m^2^)		FFMI (kg/m^2^)		FMI (kg/m^2^)		USG (au)		WC (cm)		WHR	
	Mean	sd	Mean	sd	Mean	sd	Mean	sd	Mean	sd	Mean	sd	Mean	sd	Mean	sd	Mean	sd	Mean	sd
Total sample (*n* 73)	26·2	7·3	168·1	9·0	72·0	16·8	24·7	9·1	25·3	4·7	18·9	3·3	6·4	3·2	1·018	0·005	89·3	11·0	0·85	0·05
All males (*n* 35)	26·8	8·4	173·0	7·9	80·0	16·2	20·6	7·8	26·7	4·9	21·0	3·2	5·7	3·1	1·018	0·005	90·5	10·8	0·86	0·04
All females (*n* 38)	25·6	6·2	163·6	7·4	64·7	13·8	28·5	8·5	24·0	4·2	16·9	2·0	7·1	3·2	1·018	0·005	88·3	11·3	0·85	0·05
Total sample with follow-up data (*n* 37)	28·6	8·2	168·7	8·6	72·4	15·4	22·3	7·6	25·2	3·8	19·6	3·5	5·6	2·2	1·018	0·005	88·6	8·0	0·86	0·04
Males with follow-up data (*n* 21)	30·1	9·2	172·2	8·0	80·6	14·8	20·3	7·1	27·1	3·8	21·5	3·2	5·5	2·2	1·019	0·005	91·5	8·1	0·87	0·04
Females with follow-up data (*n* 16)	26·7	6·5	164·2	7·3	61·5	7·7	25·0	7·7	22·8	2·1	17·0	1·7	5·8	2·1	1·017	0·004	84·8	6·2	0·84	0·04

FFMI, fat-free mass index from 4C model; FMI, fat mass index from 4C model; USG, urine-specific gravity; WC, waist circumference; WHR, waist–hip ratio.

### Reliability

**Fig. 1.**
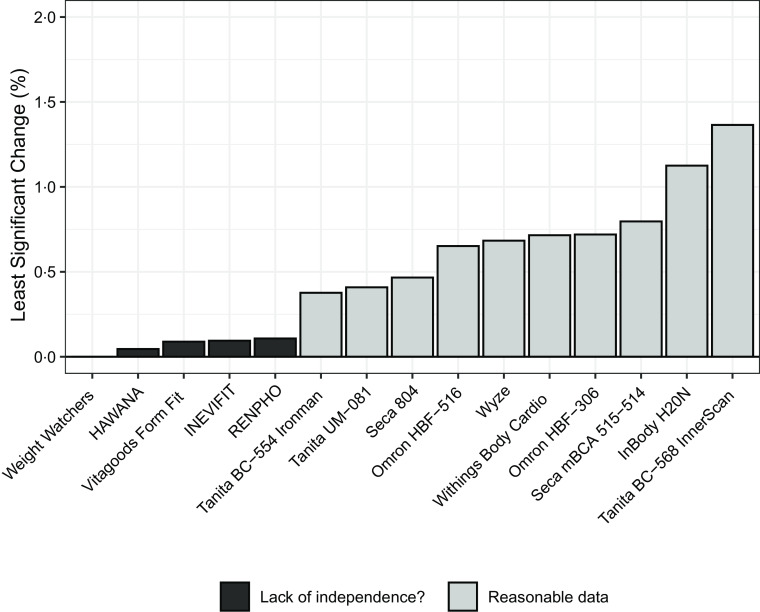
Least significant change values for body fat percentage estimates of each bioelectrical impedance analysis (BIA) device. Least significant change was calculated as 2·77 × precision error (PE, calculated as 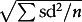 , where sd is the within-subject standard deviation), to reflect a 95 % confidence level. Least significant change ranged from 0 % to 0·11 % in five devices, indicating a potential lack of independence between data points, and from 0·38 % to 1·36 % in the remaining ten devices, indicating reasonable data.

Across all fifteen devices, the mean difference between readings ranged from −0·06 % to +0·07 %, the maximum absolute difference ranged from 0·0 % to 2·5 %, PE ranged from 0·0 % to 0·49 % and least significant change ranged from 0·0 % to 1·36 %. Notably, in a subset of five foot-to-foot devices (B, C, E, F and H), maximum absolute difference values ranged from 0·0 % to 0·1 %, accompanied by notably low values for both PE (range: 0·0–0·04 %) and least significant change (range: 0·0–0·11 %). The low observed values for these models compared with the remaining ten devices, in addition to an apparent ‘ceiling’ of observed mean differences at 0·1 %, suggest that the reliability estimates for these devices in particular should be interpreted with extreme caution. Additionally, three of the five hand-to-foot (octapolar) devices within the sample (M–O) demonstrated the highest estimates for PE and least significant change. However, for all but two (M and N) of the fifteen devices, least significant change did not exceed 0·80 %. A comparison of least significant change values across all devices is presented in Fig. 1, and a potential lack of independence among the five devices with the lowest values is noted. Detailed test–retest reliability values for each device can be found in online Supplement 2.

### Cross-sectional validity

The mean BFP estimate of the 4C model was 24·7 (sd 9·1) %. Across all devices, constant error ranged from −3·5 (sd 4·1) % to 11·7 (sd 4·7) %, standard error of the estimate ranged from 3·1 % to 7·5 %, total error ranged from 3·3 % to 12·6 % and Lin’s CCC ranged from 0·48 to 0·94. The slope of the regression line within the Bland–Altman plots ranged from −0·50 to 0·08. Six of the fifteen devices exhibited negative proportional bias and a slope that was significantly different from zero, indicating that approximately 40 % of the fifteen BIA devices investigated systematically overestimated BFP in individuals with relatively less body fat while systematically underestimating BFP in individuals with higher levels of adiposity. These devices included five foot-to-foot devices (B–D, F and H) and the lone hand-to-hand device (A). No devices demonstrated a statistically significant positive proportional bias. Only five (33 %) of the fifteen devices were statistically equivalent to the 4C model as assessed with one-sided *t* tests using a ± 2 % equivalence region; this included the lone research-grade device (O) and four consumer-grade models, including three foot-to-foot devices (G, K and L) and one octapolar model (N). A summary of the BFP estimates and select cross-sectional validity metrics is presented in Table 2.

**Table 2.** Select cross-sectional validity metrics for each device

Model	Body fat (%)					95 % CI for constant error (%)	Total error (%)	Equiv.	90 % TOST (%)	Standard error of the estimate rating*
	Mean	sd	Min	Max	CE	LL, UL			LL, UL	
Criterion (4C)	24·7	9·1	6·4	46·9	–		–	–		–
Omron HBF-306	21·2	8·0	8·5	40·6	–3·5	–4·4, −2·5	5·4	No	–4·3, −2·7	Fairly good
RENPHO Scale	23·3	7·5	6·3	45·0	–1·4	–3·1, 0·2	7·2	No	–2·8, 0·0	Poor
HAWANA Scale	27·6	6·0	8·4	41·0	2·9	1·2, 4·6	7·8	No	1·5, 4·3	Poor
Wyze Scale	28·3	6·6	8·9	46·1	3·6	2·2, 5·0	6·9	No	2·5, 4·8	Poor
INEVIFIT Scale	26·1	7·8	5·0	43·0	1·4	–0·2, 3·0	6·8	No	0·1, 2·7	Poor
Weight Watchers Scale	25·7	6·3	6·3	40·1	0·9	–0·8, 2·7	7·5	No	–0·5, 2·4	Poor
Tanita UM-081	25·3	8·8	6·5	48·0	0·6	–0·8, 1·9	5·8	Yes	–0·6, 1·7	Poor
Vitagoods Form Fit	23·7	7·3	6·9	45·6	–1·1	–2·6, 0·5	6·8	No	–2·4, 0·3	Poor
Omron HBF-516	28·0	9·6	8·3	53·6	3·3	2·3, 4·4	5·6	No	2·4, 4·2	Fair
Seca 804	36·4	9·4	20·1	67·4	11·7	10·6, 12·8	12·6	No	10·8, 12·6	Fair
Withings Body Cardio	24·4	8·7	5·1	47·5	–0·4	–1·7, 1·0	5·8	Yes	–1·5, 0·8	Poor
Tanita BC-554 Ironman	25·5	8·8	6·3	48·2	0·7	–0·6, 2·1	5·8	Yes	–0·4, 1·9	Poor
Tanita BC-568 InnerScan	23·3	9·4	6·7	48·2	–1·4	–2·4, −0·4	4·4	No	–2·2, −0·6	Fairly good
InBody H20N	24·6	9·8	5·0	50·5	–0·1	–0·9, 0·7	3·3	Yes	–0·8, 0·6	Very good
Seca mBCA 515/514	24·5	9·2	5·6	47·5	–0·2	–1·0, 0·6	3·3	Yes	–0·8, 0·4	Very good

CE, constant error; LL, lower limit; Min, minimum; Max, maximum; TOST, two one-sided *t* tests; UL, upper limit.

* Based on the subjective ranking categories of Lohman and Milliken^(26)^.

**Fig. 2.**
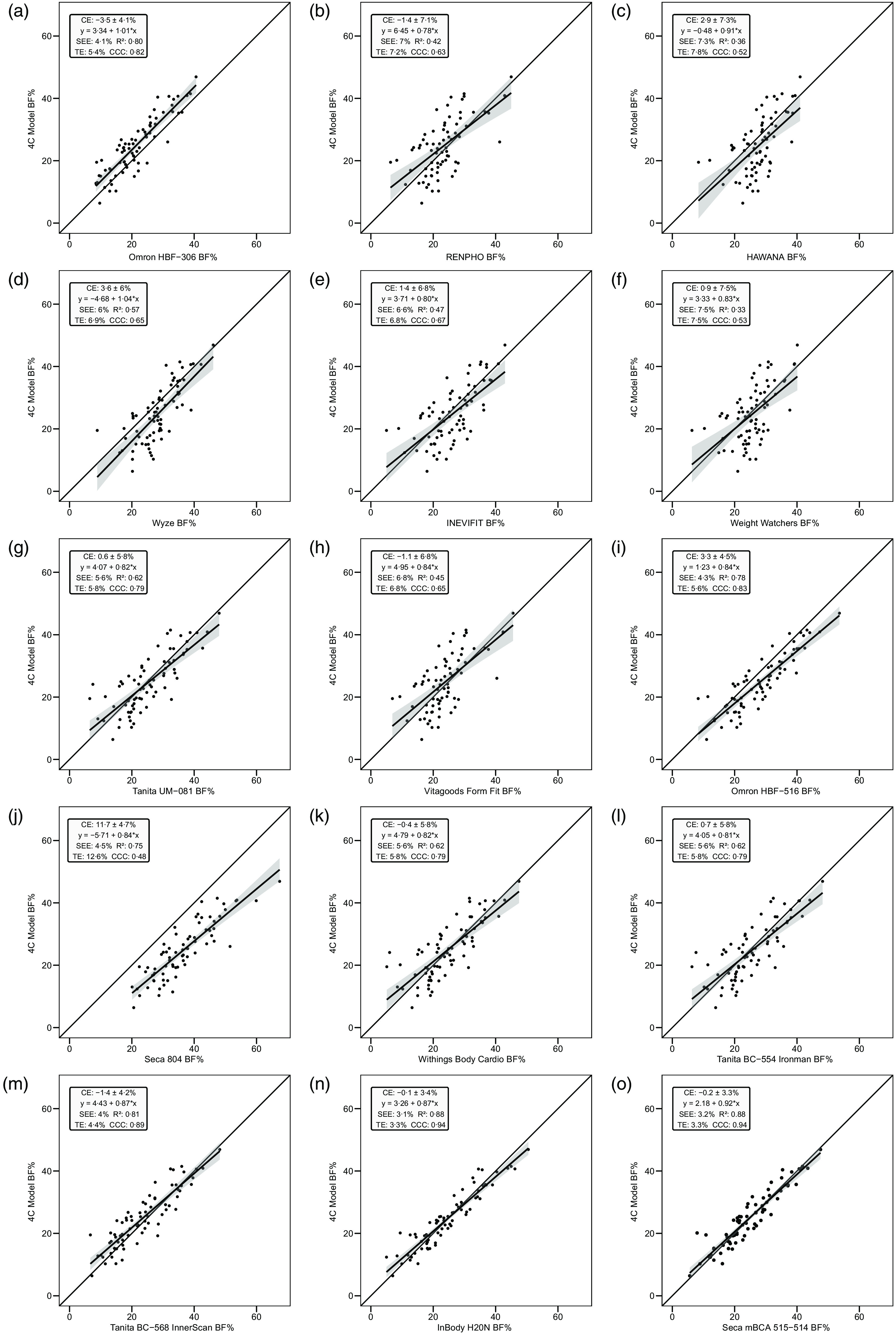
Line of identity plots for the cross-sectional validity of body fat percentage (BFP) estimates of each bioelectrical impedance analysis (BIA) device compared with the four-compartment (4C) model. Plots A–O depict ordinary least squares (OLS) regression lines for the performance of each BIA device in estimating BFP (*x*-axis) as compared with the line of identity, which represents perfect agreement with the 4C model estimate (*y*-axis). The shaded regions indicate the 95 % confidence limits for the OLS regression line. Constant error (CE), standard error of the estimate (SEE), coefficient of determination (*R* ^2^), total error (TE) and Lin’s concordance correlation coefficient (CCC) are also displayed for each device. 4C, four-compartment model; BF%, body fat percentage.

**Fig. 3.**
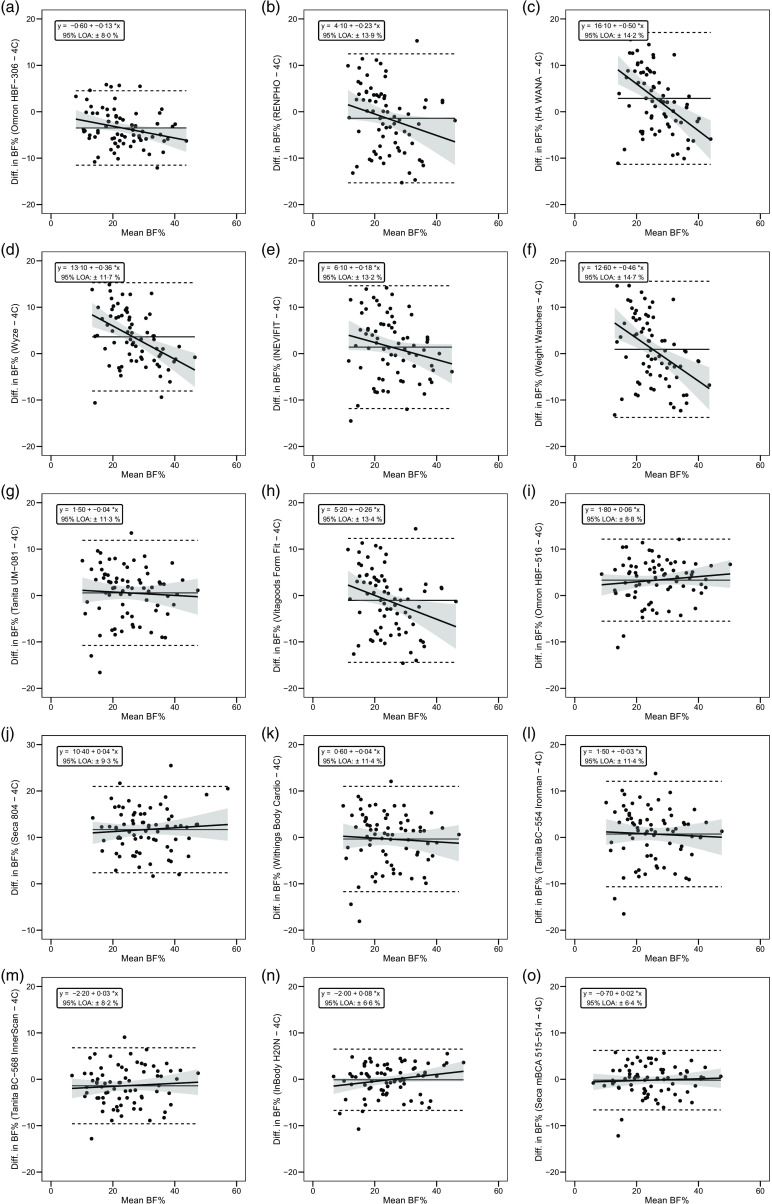
Bland–Altman plots for the cross-sectional validity of body fat percentage (BFP) estimates of each bioelectrical impedance analysis (BIA) device compared with the four-compartment (4C) model. Plots A–O show the relationship between the average of the BFP estimates of each BIA device and the reference 4C model (*x*-axis) and the difference in the estimate from the BIA device minus that of the 4C estimate (*y*-axis). The shaded regions around the diagonal line indicate the 95 % confidence limits for the linear regression line, the horizontal dashed lines indicate the upper and lower limits of agreement (LOA) and the horizontal solid line indicates the constant error between methods. Linear regression equations and 95 % LOA values are also displayed. 4C, four-compartment model; BF%, body fat percentage.

Overall, a subset of the foot-to-foot devices tended to perform more poorly across metrics compared with octapolar models. However, one foot-to-foot device in particular (J) demonstrated relatively poor cross-sectional validity compared with the other devices (constant error: 11·7 %; total error: 12·6 %; CCC: 0·48). Excluding this model, the maximum constant error across the remaining fourteen devices was 3·6 % and the maximum total error was 7·8 %. Figure 2 shows the line of identity plots for the cross-sectional validity of BFP estimates of each BIA device as compared with those of the 4C model using OLS regression, and Fig. 3 presents the Bland–Altman plots for the cross-sectional validity of these devices. Detailed cross-sectional validity values, including the results of both OLS and Deming regressions, can be found in online Supplement 3.

### Longitudinal validity

During the 12–16 weeks between the initial and optional follow-up visit, the mean change in BFP as assessed with the 4C model was 0·2 (sd 2·9) %. Across participants, change in BFP within the 4C model ranged from −5·0 % to 7·5 %, with 19/37 (51 %) participants exhibiting an increase in BFP and 18/37 (49 %) exhibiting a decrease. Across the sample of BIA devices, mean observed change over time ranged from −0·2 (sd 2·3) % to 1·4 (sd 3·6) %, and constant error ranged from −0·4 (sd 2·1) % to 1·3 (sd 2·7) %. Standard error of the estimate ranged from 1·7 % to 2·6 %, total error ranged from 1·9 % to 2·9 % and Lin’s CCC ranged from 0·37 to 0·78. Nine (60 %) of the devices were statistically equivalent to the 4C model based as assessed with one-sided *t* tests using a ± 1 % equivalence region. A summary of the BFP estimates and select longitudinal validity metrics is presented in Table 3.

**Table 3.** Select longitudinal validity metrics for each device

Model	Change in body fat (%)					95 % CI for constant error (%)	Total error (%)	Equiv.	90 % TOST (%)
	Mean	sd	Min	Max	CE	LL, UL			LL, UL
Criterion (4C)	0·2	2·9	–5·0	7·5	–		–	–	
Omron HBF-306	0·4	2·0	–4·1	6·1	0·2	–0·5, 0·9	2·1	Yes	–0·4, 0·8
RENPHO Scale	–0·1	1·3	–3·9	3·2	–0·3	–1·1, 0·6	2·5	Yes	–1·0, 0·4
HAWANA Scale	0·1	1·5	–2·4	5·9	0·0	–0·9, 0·8	2·6	Yes	–0·8, 0·7
Wyze Scale	0·4	2·2	–3·6	7·3	0·3	–0·4, 1·0	2·1	Yes	–0·3, 0·9
INEVIFIT Scale	0·2	1·6	–2·6	6·5	0·0	–0·7, 0·8	2·2	Yes	–0·6, 0·7
Weight Watchers Scale	–0·1	1·4	–2·8	3·8	–0·2	–1·1, 0·6	2·5	Yes	–0·9, 0·5
Tanita UM-081	0·8	2·5	–3·9	10·3	0·7	0·0, 1·3	1·9	No	0·1, 1·2
Vitagoods Form Fit	–0·1	1·3	–3·8	3·0	–0·3	–1·1, 0·6	2·5	Yes	–1·0, 0·4
Omron HBF-516	1·0	2·6	–3·8	9·7	0·8	0·2, 1·4	1·9	No	0·3, 1·3
Seca 804	1·4	3·6	–4·2	10·0	1·3	0·4, 2·2	2·9	No	0·5, 2·0
Withings Body Cardio	0·8	2·6	–3·6	10·0	0·6	0·0, 1·2	1·9	No	0·1, 1·1
Tanita BC-554 Ironman	0·8	2·4	–3·5	9·3	0·6	0·0, 1·3	2·0	No	0·1, 1·2
Tanita BC-568 InnerScan	0·9	2·6	–4·0	7·6	0·7	0·1, 1·3	1·9	No	0·2, 1·2
InBody H20N	–0·2	2·3	–5·2	4·8	–0·4	–1·0, 0·3	2·1	Yes	–0·9, 0·2
Seca mBCA 515/514	0·5	2·5	–4·3	6·8	0·3	–0·4, 1·0	2·1	Yes	–0·3, 0·9

CE, constant error; LL, lower limit; Min, minimum; Max, maximum; TOST, two one-sided *t* tests; UL, upper limit.

**Fig. 4.**
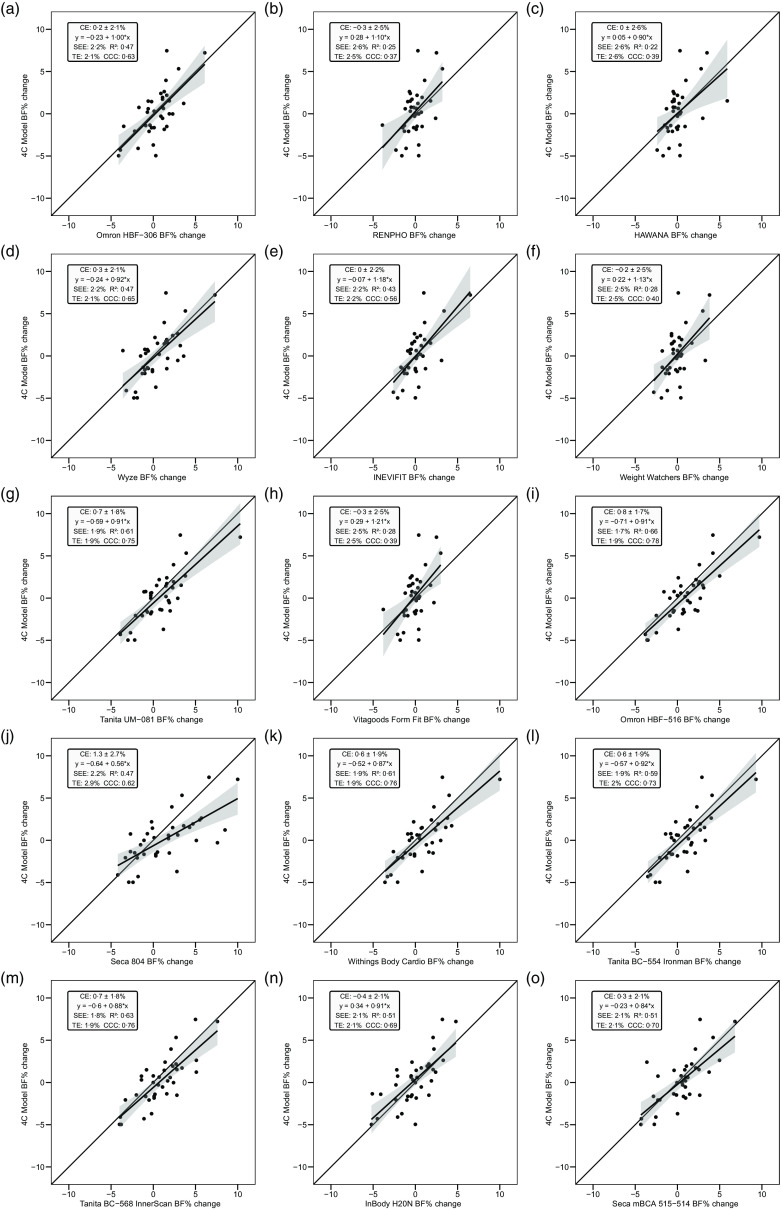
Line of identity plots for the longitudinal validity of body fat percentage (BFP) estimates of each bioelectrical impedance analysis (BIA) device compared with the four-compartment (4C) model. Plots A–O depict ordinary least squares (OLS) regression lines for the performance of each BIA device in estimating change in BFP (*x*-axis) as compared with the line of identity, which represents perfect agreement with the 4C model estimate (*y*-axis). The shaded regions indicate the 95 % confidence limits for the OLS regression line. Constant error (CE), standard error of the estimate (SEE), coefficient of determination (*R* ^2^), total error (TE) and Lin’s concordance correlation coefficient (CCC) are also displayed for each device. 4C, four-compartment model; BF%, body fat percentage.

**Fig. 5.**
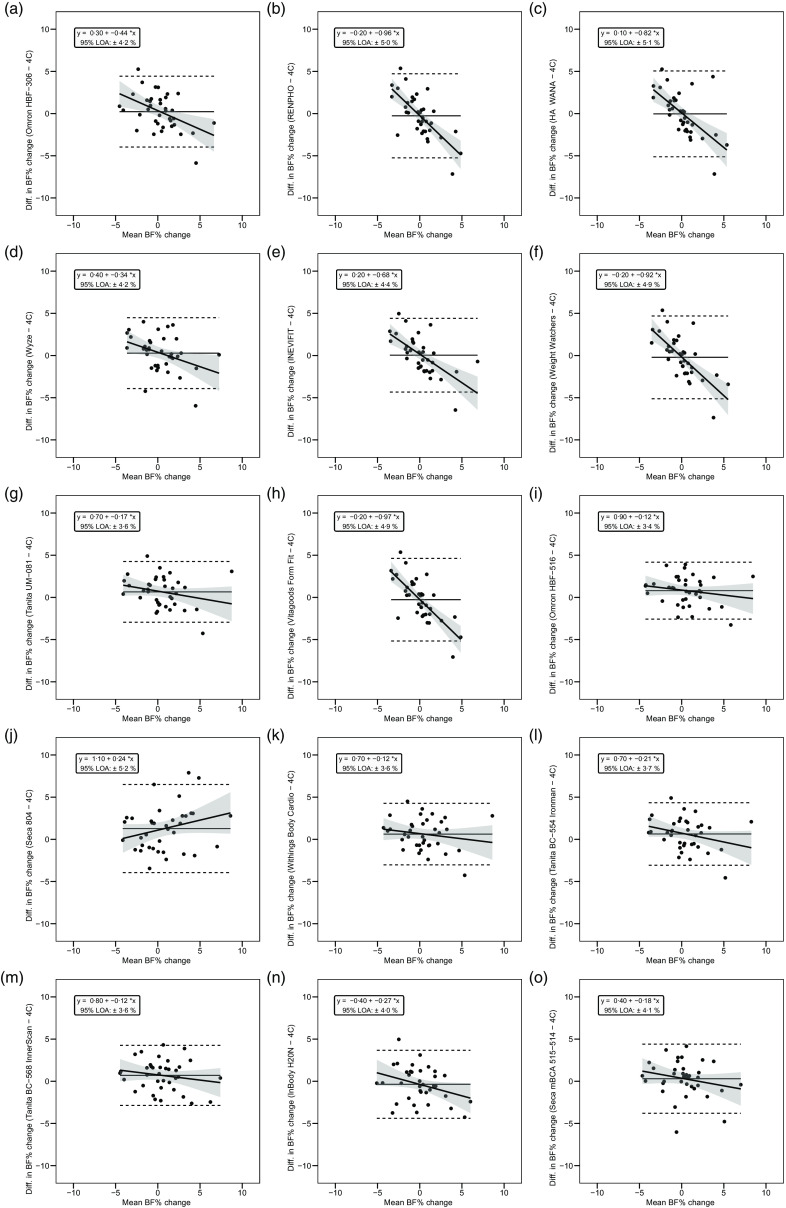
Bland–Altman plots for the longitudinal validity of body fat percentage (BFP) estimates of each bioelectrical impedance analysis (BIA) device compared with the four-compartment (4C) model. Plots A–O show the relationship between the average of the change in BFP estimated by each BIA device and by the reference 4C model (*x*-axis) and the difference in change estimates from the BIA device minus that of the 4C estimate (*y*-axis). The shaded regions around the diagonal line indicate the 95 % confidence limits for the linear regression line, the horizontal dashed lines indicate the upper and lower limits of agreement (LOA) and the horizontal solid line indicates the constant error between methods. Linear regression equations and 95 % LOA values are also displayed. 4C, four-compartment model; BF%, body fat percentage.

The slope of the regression line of the Bland–Altman plot ranged from −0·97 to 0·24. The Bland–Altman slope was significantly different from zero in seven (47 %) of the fifteen devices, all of which demonstrated negative proportional bias. These models included six foot-to-foot devices (B–F and H) and the lone hand-to-hand device (A). The presence of negative proportional bias indicated that the BIA devices systematically produced more numerically positive BFP change values, as compared with 4C, in individuals with more numerically negative BFP changes while systematically producing more numerically negative BFP change values, as compared with 4C, in individuals with more numerically positive BFP changes. As with cross-sectional validity, no devices exhibited positive proportional bias to a statistically significant degree. In addition, a subset of foot-to-foot devices generally performed the most poorly across all metrics, with octapolar models generally performing among the top half of devices. Figure 4 shows the line of identity plots for the longitudinal BFP estimates of each BIA device as compared with those of the 4C model using OLS regression, and Fig. 5 shows the Bland–Altman plots for the longitudinal validity of these devices. Detailed cross-sectional validity values, including the results of both OLS and Deming regressions, can be found in online Supplement 4.

### Global performance of devices across metrics

Table 4 presents relative rankings of each device in terms of test–retest reliability, cross-sectional and longitudinal group validity, cross-sectional and longitudinal individual validity and global performance score. Due to the suspected lack of independence within the test–retest reliability data of five devices (B, C, E, F and H), these devices were ranked in the lowest place for reliability (i.e. tied for 11th place).

**Table 4.** Device test–retest reliability, cross-sectional validity, longitudinal validity and global performance scores and ratings

Model	Global ranking	Global sum	Group-level validity rankings		Individual-level validity rankings		Reliability ranking
			Cross-sectional	Longitudinal	Cross-sectional	Longitudinal	
Omron HBF-516	1	16	5	1	5	1	4
Tanita BC-568 InnerScan	2	21	3	2	4	2	10
Tanita UM-081	3	22	7	3	7	3	2
Seca mBCA 515/514	4	24	1	7	1	7	8
InBody H20N	5	25	2	6	2	6	9
Withings Body Cardio	6	28	6	4	8	4	6
Tanita BC-554 Ironman	6	28	8	5	9	5	1
Omron HBF-306	8	30	4	8	3	8	7
Wyze Scale	9	44	11	9	10	9	5
INEVIFIT Scale	10	52	10	10	11	10	11
Seca 804	11	54	15	15	6	15	3
Vitagoods Form Fit	12	55	9	12	12	11	11
RENPHO Scale	13	62	12	13	13	13	11
Weight Watchers Scale	13	62	13	11	15	12	11
HAWANA Scale	15	67	14	14	14	14	11

## Discussion

The purpose of the present study was to expand upon the limited existing research on consumer-grade BIA devices through a thorough evaluation of the reliability, cross-sectional validity and longitudinal validity of fifteen popular BIA devices.

### Reliability

While test–retest reliability among the sample of BIA devices varied, it may generally be considered acceptable for the devices investigated given their common application in consumer and field settings. However, five foot-to-foot devices within our sample demonstrated suspiciously high reliability values in the context of immediate retesting. Thus, these models may include within their algorithms a feature designed to prevent the generation of multiple outputs with differences that exceed a certain limit (e.g. 0·1 %) if multiple readings are taken within a designated period of time (e.g. within several minutes of one another) and while the same user account (i.e. individual information entered and saved within the device or accompanying mobile application) is being accessed.

It is possible that manufacturers develop these restrictions in order to improve consumers’ perception of the accuracy of a device when accessed by the same user; however, our results indicate a lack of independence between immediately adjacent tests. The use of a ‘guest mode’ feature available on other devices within our sample may have avoided this pitfall and allowed for the generation of more apparently independent data between repeated tests from those devices. However, given that consumers’ use of these devices typically includes the creation of an individual account in which user data are saved for repeated tests, consumers should be aware that immediate reuse of certain BIA devices may provide the same estimates within a given time period.

On the other hand, three of the five octapolar devices (M–O) demonstrated the highest values for PE and least significant change. This may suggest that the addition of hand-to-hand components in these devices may introduce error generated either by the user (i.e. the additional error introduced by the variable placement of hands and arms) or by the greater amount of bioelectrical data measured by the device (i.e. the measurement of reactance and resistance throughout the tissues of all four limbs as opposed to only two). Taken together, these findings suggest that reliability metrics taken at face value likely do not provide a full picture of the accuracy or utility of a given BIA device for the typical consumer and should instead be interpreted within an informed context. For instance, very low PE values may actually indicate that the device is not providing a true second estimate if tests are taken in immediate succession; meanwhile, a device with PE that is within a higher but reasonable range of values may actually indicate that it is more sensitive to small shifts in user foot/hand position, posture or other factors and is thus using more data to inform each individual estimate.

Despite the nuances of interpreting reliability values in the context of BIA devices, least significant change can provide some insight to the sensitivity of each device in detecting physiological changes related to body composition at the individual level. Within the current sample, least significant change for all devices did not exceed 1·4 %, and for all but two of the devices within our sample did not exceed 0·8 %. At face value, these data indicate that a user may generally be confident that, within the range of devices investigated in this study, an observed change of at least 1·4 % in either direction indicates that real physiological change in body composition has occurred, provided that the same pre-assessment standardisation procedures described in this report are implemented. However, it should be noted that reliability metrics were produced using immediate test–retest reliability, indicative of technical precision, which does not account for day-to-day biological variability. Accordingly, calculation of the same reliability metrics using values from separate days would almost certainly produce higher error rates^(25)^. In the present investigation, the question of whether bioimpedance techniques can assess real changes in body composition across multiple days was addressed directly through longitudinal validity testing.

### Cross-sectional validity

Six (40 %) of the fifteen devices in our sample demonstrated statistically significant negative proportional bias as demonstrated in the Bland–Altman analysis. In other words, many of these devices demonstrate a lack of sensitivity required to accurately estimate BFP in individuals on either end of a population’s distribution while generally exhibiting greater accuracy in estimating BFP in those with intermediate values. This may point to an artificially restricted range of output values programmed in these devices based on a manufacturer’s desire to provide a relatively ‘normal’ BFP estimate to a consumer. It may also indicate the use of an exceedingly homogenous reference population by the manufacturer when developing algorithms. Therefore, those with levels of body fat further from the population norm should generally be more sceptical of outputs received by a given consumer-grade BIA device.

### Longitudinal validity

Generally, a subset of the foot-to-foot devices tended to perform the most poorly while the octapolar models (including one research-grade and three consumer-grade models) tended to perform among the top half of devices. Approximately half (47 %) of the investigated models demonstrated statistically significant negative proportional bias as assessed with Bland–Altman analysis. These devices, including six foot-to-foot and one hand-to-hand device, also tended to exhibit lower longitudinal concordance and correlation values (i.e. CCC, *r*, *R* ^2^) and higher error (i.e. standard error of the estimate and total error). This indicates that a considerable portion of devices within our sample could not adequately detect changes in body composition over time as compared with the reference 4C model.

Overall, the relatively poor longitudinal validity demonstrated in approximately half of our sample of devices indicates that consumers should be wary of the ability of such devices to accurately track changes in body composition over time, at least on the population level. On the other hand, there was high variability in performance across devices, and several models (notably devices G, I and K–O) performed comparably to the research-grade device within our sample as compared with the changes detected by the 4C criterion model. Thus, select models of BIA devices may hold potential utility for the tracking of body composition over time, particularly in contexts in which the purchase or use of a research-grade device is infeasible. Generally, octapolar models and select foot-to-foot devices tended to demonstrate higher longitudinal validity than the other models within the sample.

### Strengths and limitations

Strengths of this study include the wide range of popular BIA devices investigated, the inclusion of pre-assessment standardisation protocols which limited the amount of error introduced by hydration status and other factors and the assessment of the longitudinal validity of devices in tracking changes in body composition over 12–16 weeks in a free-living population. However, some potential error may have been introduced by the conversion and rounding of inputs required for the use of certain devices, although this error was likely minimal (e.g. differences between measured and resulting height inputs not to exceed approximately 0·25 inches or 0·6 cm for device A). For one device (D), a firmware update occurred during the initial data collection period, and this update was confirmed to include a change to the algorithm. However, assessment of longitudinal validity values for this device appeared comparable to those of devices with a similar type (i.e. foot-to-foot) and price range. Additionally, the scales ranged in terms of their ease of use, and the complexity of use likely increased with the addition of hand-to-hand elements in octapolar models. Though each participant was instructed by research staff on the proper use of each device, it is possible that the conformation of hand and/or foot placement on a given device, and thus the reliability of its resulting estimates, may increase as an individual becomes more familiar with each model.

Another consideration is the possibility that devices demonstrating lower error in the present investigation could have been validated in samples similar to that of the present study, unlike those demonstrating poorer performance, although this is speculative due to the lack of accessible information regarding the development and validation of analysers used in this study. Nonetheless, the characteristics of the present sample should be considered before indiscriminately assuming equivalent performance in other groups. Finally, although a ranking system was developed to enable interpretation of the performance of each device across a range of potential domains of interest, these rankings were based on singular metrics within each domain (e.g. total error as a general surrogate for group-level cross-sectional and longitudinal validity) and thus represent a simplified illustration of the reliability and validity of each model.

### Conclusion

Though test–retest reliability as well as cross-sectional and longitudinal validity values varied across the sample of BIA devices investigated, some models demonstrated adequate performance across several domains. Thus, the utility of this class of BIA devices for the accurate assessment of body composition values largely depends upon the model in question.

### Suggested best practices for use of consumer-grade BIA devices

Importantly, use cases for commercially available, consumer-grade BIA devices are not just limited to consumers who choose to purchase BIA devices for personal use. End users may also include proprietors of recreational facilities as well as researchers, coaches and other practitioners looking to assess body composition in athletes, clientele or other individuals in settings in which the use of research-grade devices is infeasible. Given the wide-ranging potential applications of BIA devices, it is important that consumers and other users are aware of best practices to improve the utility of these models. These suggestions include: (1) following existing pre-assessment standardisation protocols whenever possible, such as assessing body composition each morning immediately after an overnight fast from food and fluid, after voiding and while wearing minimal clothing; (2) tracking body composition values on a multi-day average basis rather than using singular estimates and (3) using the same device to track longitudinal changes in body composition, with interpretation of change scores informed by the observed reliability of that device or similar devices investigated in this report.

In addition to these suggestions, the use of octapolar models over foot-to-foot or hand-to-hand devices will likely improve the validity of resulting estimates in both cross-sectional and longitudinal contexts. While the higher complexity of these devices may result in lower apparent reliability of values on a test-to-test basis, averaging duplicate assessments on a given day – and subsequently using these averages in the calculation of a multi-day average – is expected to ameliorate this issue.

### Suggestions for future research

Future research should expand upon previous investigations of the effect of various activity level inputs allowed by devices (e.g. ‘normal’ *v*. ‘athlete’ mode) on both cross-sectional and longitudinal validity and based on various definitions of activity levels to determine these inputs. In addition, our findings warrant further investigation of an apparent ‘ceiling’ of differences between consecutive readings on certain devices, which may result in a lack of independence between readings upon immediate retesting in these models. As demonstrated, the utility of consumer-grade BIA analysers should ideally be considered on a case-by-case basis. Therefore, the continued examination of new analysers is necessary to better evaluate their utility in diverse settings. Additionally, manufacturers of BIA analysers should invest in appropriate research and development activities to ensure they produce devices with the requisite accuracy to benefit users.

## Supplementary material

For supplementary material accompanying this paper visit https://doi.org/10.1017/S0007114522003749. click here to view supplementary material
